# Novel Aminosilane (APTES)-Grafted Polyaniline@Graphene Oxide (PANI-GO) Nanocomposite for Electrochemical Sensor

**DOI:** 10.3390/polym13152562

**Published:** 2021-07-31

**Authors:** Raja Saad Alruwais, Waheed A. Adeosun, Hadi M. Marwani, Mohammad Jawaid, Abdullah M. Asiri, Anish Khan

**Affiliations:** 1 Chemistry Department, Faculty of Science, King Abdulaziz University, Jeddah 21589, Saudi Arabia; rajaotb@gmail.com (R.S.A.); dsnwaheed1@gmail.com (W.A.A.); hmarwani@kau.edu.sa (H.M.M.); aasiri2@kau.edu.sa (A.M.A.); 2 Chemistry Department, Faculty of Science and Humanities, Shaqra University, Dawadmi 11911, Saudi Arabia; 3 Center of Excellence for Advanced Materials Research, King Abdulaziz University, Jeddah 21589, Saudi Arabia; 4 Laboratory of Biocomposite Technology, Institute of Tropical Forestry and Forest Products (INTROP), University Putra Malaysia, UPM Serdang 43400, Selangor, Malaysia

**Keywords:** linear sweep voltammetry, heavy metal detection, environmental pollution, lead ion detection, electrochemical sensors, potential toxic metals

## Abstract

Lead is a potentially toxic element (PTE) that has several adverse medical effects in humans. Its presence in the environment became prominent due to anthropogenic activities. The current study explores the use of newly developed composite materials (organic–inorganic hybrid) based on PANI-GO-APTES for electrochemical detection of Pb^2+^ in aqueous solution. The composite material (PANI-GO-APTES) was synthesized by chemical method and was characterized with SEM, XPS, XEDS, XRD, TGA, FTIR, EIS and CV. The result of characterization indicates the successful synthesis of the intended material. The PANI-GO-APTES was successfully applied for electrochemical detection of Pb^2+^ using cyclic voltammetry and linear sweep voltammetry method. The limit of detection of Pb^2+^ was 0.0053 µM in the linear range of 0.01 µM to 0.4 µM. The current response produced during the electrochemical reduction of Pb^2+^ catalyzed by PANI-GO-APTES was also very repeatable, reproducible and rapid. The application of PANI-GO-APTES-modified GCE in real sample analysis was also established. Therefore, PANI-GO-APTES is presented as a potential Pb^2+^ sensor for environmental and human health safety.

## 1. Introduction

Hybrid organic–inorganic (HOI) composites were discovered several decades back. However, they did not attract the interest of the scientific community until the 1990s when Toyota recorded a massive increase in the strength of the materials used for its automobiles by incorporation of mica to nylon to yield novel HOI material [1,2]. HOI materials have diverse applications in many fields, including automobiles, textiles and aircraft. Often, the inorganic component of the HOI contributes some properties such as magnetic and dielectric effects and chemical and thermal stability [3,4,5]. On the other hand, the organic component ensures properties such as low density, tenability and luminescence [3,4,5]. The use of 2D materials as components of HOI has gained much attention because it delivers appealing hybrid materials [6]. Lately, most studies have focused on the use of 2D materials based on carbon, metal oxide and even polymers to fabricate HOI. Many conducting polymers (e.g., polyaniline, polypyrrole, poly-o-toluidine) fit in this role very well. Specifically, polyaniline/graphene hybrids synthesized by the chemical oxidative method exhibit excellent electrical conductivity, high specific capacitance and mechanical stability [7,8,9]. 

The unrivaled excellent properties of graphene and graphene oxide such as large surface area, high electrical conductivity, high potential adsorption power and good catalytic properties make them efficient materials for several applications, including supercapacitors, energy storage devices and electrochemical sensing [10,11,12,13,14,15,16]. In addition, silane-based materials have been reported previously to improve electrical conductivity and chemical stability of materials [17,18,19]. Therefore, the composition of polyaniline (PANI), graphene oxide (GO) and 3-aminopropyl-triethoxylsilane (APTES) is expected to yield an effective electrocatalyst that is able to catalyze electro-redox processes such as electroreduction of toxic metals in aqueous solution. Among the most dominant toxic metals in the environment is lead [20]. 

Lead is primarily introduced into the environment by industrial activities such as industrial operations at mining sites. Other sources include lead battery manufacturers, lead-based pigment manufacturers, automobile industries, decayed water-carrying metal pipes and contaminated consumer products [20]. Exposure to lead at a certain concentration has been implicated with several health problems such as renal dysfunction, nervous system impairment, headache, fatigue, constipation and brain development impairment in children [21,22,23,24,25,26,27,28].

Exposure to lead has been implicated in an average of 143,000 deaths annually [20]. Because of its adverse effects on human health, several regulatory bodies including World Health Organization (WHO) have pegged the permissible limit of Pb in drinking water to 15 ppb (72 nM) [29]. The Environmental Protection Agency (EPA) has stressed that one-fifth of lead exposure originates from drinking contaminated water [30]. Therefore, there is a need for detection and monitoring of lead concentration in drinking water, surface water and treated industrial effluents before emptying/discharging into rivers.

The conventional methods for toxic metals, including Pb^2+^ detection in environmental samples. Include the atomic absorption spectrophotometry method and the inductively coupled plasma method. The high cost of instrumentation and cumbersome sample preparation limit the use of the aforementioned methods. Other methods including UV-Vis spectroscopy and fluorescence spectroscopy have been used [31,32,33,34]. However, these methods also suffer from cumbersomeness in sample preparation and slow analysis time. The electrochemical method is another method not yet fully explored. The electrochemical method offers fast analysis time, low cost and little or no sample preparation [32,35]. Several studies have been conducted on electrochemical detection of lead (II) ion. For instance, in the studies conducted by Rahman et al. [36], lead (II) ion was determined in aqueous solution using the cyclic voltammetric method. The mechanism of lead detection was based on adsorption on a hanging mercury drop electrode. The detection limit of lead (II) ion exhibited by this method was 3.55 µM in the linear dynamic range of 0.02–1.0 mM. Although this procedure has a relatively wide linear range, the reported limit of detection was too high. Moreover, the toxicity of the mercury electrode may limit the potential application of this procedure for real-life analysis. In another study by Riyanto [37], cyclic voltammetry method was equally employed for the determination of lead (II) ions in industrial wastewater. The author used platinum wire as the working electrode for the detection of lead (II) ions. The limit of detection of lead (II) ion was 0.902 mg/L in the linear dynamic range of 10–70 mg/L. While the procedure exhibited a low limit of detection, the choice of working electrode based on noble metal (Pt wire), which is not only expensive but also very scarce, makes this procedure less attractive. In a recent study by Magerusan et al. [38], nitrogen-doped graphene/chitosan nanocomposite was successfully applied for lead (II) ion sensing in water. The developed method yielded a low limit of detection (0.066 µM) with a wide linear range. However, added interfering species such as Ni^2+^ and Cu^2+^ had an influence on the current response of the working electrode towards lead (II) ion especially at high concentration. Anambiga and his coresearchers worked on lead (II) ion detection using a glutathione–silver-modified screen-printed electrode that employed the electrochemical method [39]. The authors reported a lead (II) ion limit of detection of 0.049 µM. However, the authors did not validate the efficacy of their method in real water samples. In our current study, the choice of the working electrode was carefully based on cost, availability and toxicity. The materials selected for the working electrode are cheap and readily available and have very low or no toxicity. Unlike the earlier reported methods, the current study exhibited a very low limit of detection, high sensitivity and stability in the presence of likely interferents. In addition, this current study validated the efficacy of PANI-GO-APTES-modified glassy carbon electrode for lead (II) ion detection in real water samples. Therefore, the aim of this study was to develop a cheap, accurate and sensitive electrochemical-based method for lead detection in water. 

In this study, GO-APTES composite was formed by covalent interaction with 3-aminopropyltriethoxysilane (APTES) through the chemical method. The obtained GO-APTES was then integrated into the PANI matrix precursor to form an organic–inorganic hybrid. The as-prepared PANI-GO-APTES was then applied as a lead electrochemical sensor at trace concentration. This study, as far as we know, reports for the first time the synthesis of the composite under study (PANI-GO-APTES) and its use for the detection of lead ions in aqueous solution.

## 2. Materials and Methodology

### 2.1. Reagents

The reagents used for this study, namely graphene oxide, aniline monomer, potassium persulfate (PPS), 3-aminopropyltriethoxysilane (APTES, 98%), hydrochloric acid, ethanol (EtOH), acetate buffer, lead sulfate, nickel chloride, copper sulfate, cadmium sulfate, manganese sulfate, sodium nitrite, sodium hydrogen phosphate and sodium hydroxide, were supplied by Sigma-Aldrich (St. Louis, MO, USA) and Alfa Aesar (Karlsruhe, Germany). The reagents were used for the study as received. The reagents were prepared using deionized water (18.6 MΩ cm^−1^). 

### 2.2. Instrumentation

This study was carried out with the aid of the following instruments: scanning electron microscope (JEOL JSAM 6300, Jeol, Tokyo, Japan), energy-dispersive X-ray spectroscopy system (X-Max Oxford, Oxford Instruments, Abingdon, UK), Fourier transform infrared spectrometer (Perkin Elmer 2000, Waltham, MA, USA), Sonicator (Elma Sonic P180H, Elma Schmidbauer GmbH, Mannheim, Germany), thermogravimetric analyzer (Mettler Toledo, Greifensee, Switzerland), X-ray diffractometer (Thermo Scientific, Waltham, MA, USA), X-ray photoelectron spectroscope (Thermo Scientific, Waltham, MA, USA) and 4-probe conductivity meter (DMV-001, SES Instrumentation PVT. LTD., Roorkee, India) electrochemical workstation (Autolab AUT85587, Utrecht, The Netherlands).

### 2.3. Procedure for Preparation of Polyaniline (PANI)

In this study, wet chemical polymerization method was used for PANI synthesis. The typical procedure involved the preparation of three different PANI samples by mixing aniline (varying concentrations) and oxidizing agent (PPS) in a ratio of 1:3 under continuous stirring for two hours until the color of the suspension changes to green.

### 2.4. Procedure for Preparation of GO-APTES

Two hundred milligrams of GO was dispersed in ethanolic solution (3:2 *v*/*v*) under sonication for 1 h in standard flask. After a homogenous suspension of GO has been achieved, 2 mL of APTES was added to the GO dispersion and allowed to interact. The mixture GO-APTES was then refluxed for 24 h maintaining 70 °C temperature.

### 2.5. Introduction of PANI Matrix into GO-APTES Composite

**Scheme 1:**
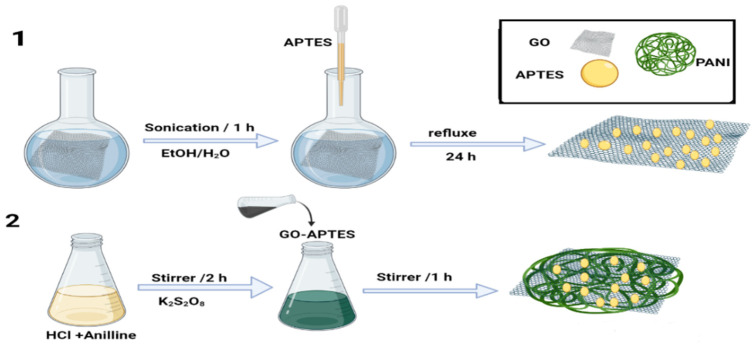
Diagrammatic illustration of PANI-GO-APTES synthesis.

The GO-APTES mixture was added to PANI in a 500 mL flask with continuous stirring for 1 h, after which the mixture was kept in the refrigerator for 3 h. The obtained mixture of PANI-GO-APTES was filtered using Whatman filter paper. The residue was washed thoroughly with double-distilled water to remove impurities. The purified residues were then dried overnight and collected in a clean mortar where they were ground into powder form. The as-prepared PANI-GO-APTES was then stored and used for further studies. The whole synthetic procedure is illustrated in Scheme 1.

### 2.6. Characterization of the Synthesized Sample (PANI-GO-APTES)

The techniques employed for characterizing PANI-GO-APTES included SEM, XRD, XPS, TGA, FTIR and cyclic voltammetry.

SEM was used for the investigation of the morphology of the as-prepared PANI-GO-APTES. The SEM images were collected at low and high magnifications. XRD was used for structural analysis using Cu K radiation (λ = 0.15418 nm) in the 2-theta angle range of 10–80° and scan rate of 0.05°/min. The elemental composition was investigated using XPS and EDX. For the XPS analysis, Al K monochromatic X-radiation source was used with a beam spot of 300 µm. EDX was fitted with SEM and simultaneously recorded with SEM imaging.

FTIR analysis was conducted to reveal the functionalities of the as-prepared PANI-GO-APTES. The spectra were collected at the IR wavenumber of 400–4000 cm^−1^ using KBr pellets.

TGA analysis was carried out to reveal information about the thermal stability of the synthesized sample. It was carried out in a nitrogen atmosphere in the heating range of 50–900 °C and heating rate of 20 °C/min and a gas flow of 20 mL/min. 

Electrical conductivity of the as-prepared PANI-GO-APTES was measured using a 4-probe conductivity meter. The as-prepared PANI-GO-APTES was made into pellets using a presser by applying a pressure of 5 kN with hydraulic pressure of 20 min. The produced pellets were then used for conductivity measurements.

Electrochemical characterization and measurements were carried out using Autolab potentiostat. The typical potentiostat contains three electrodes: working, reference and counter electrodes. The working electrode was either a bare glassy carbon electrode (GCE) or PANI-GO-APTES-modified GCE at any point in time in this study. The reference electrode was a silver/silver chloride electrode system, and the counter electrode was a platinum wire. 

### 2.7. Electrochemical Detection of Lead (II) Ions

Detection of lead ions was carried out by electrochemical means. Specifically, cyclic voltammetry and linear sweep voltammetry methods were used. For the CV study, the potential window ranged from −1.2 to 1.0 V, using a scan rate of 75 mV/s and amplitude of 0.005 V. The linear sweep was used for calibration study and was carried out maintaining the potential window range of −0.8 to 0 V at a scan rate of 75 mV/s.

### 2.8. Fabrication of Pb^2+^ Electrochemical Sensor Probe

The designed electrochemical sensor probe for Pb^2+^ was based on PANI-GO-APTES-modified GCE. To start with, the bare electrode was thoroughly washed with distilled water and ethanol and electrochemically cleaned in 0.25 M H_2_SO_4_ (by CV). To the clean bare GCE, 10 µg of PANI-GO-APTES powdery material was dispersed in 10 µL Nafion solution. The dispersed PANI-GO-APTES/Nafion solution was then cast on the GCE and was allowed to anneal in the oven for about 10 min. The PANI-GO-APTES-GCE was then stored and used for further studies.

## 3. Results and Discussions

### 3.1. Results of Characterization

#### 3.1.1. Morphological Investigation

**Figure 1:**
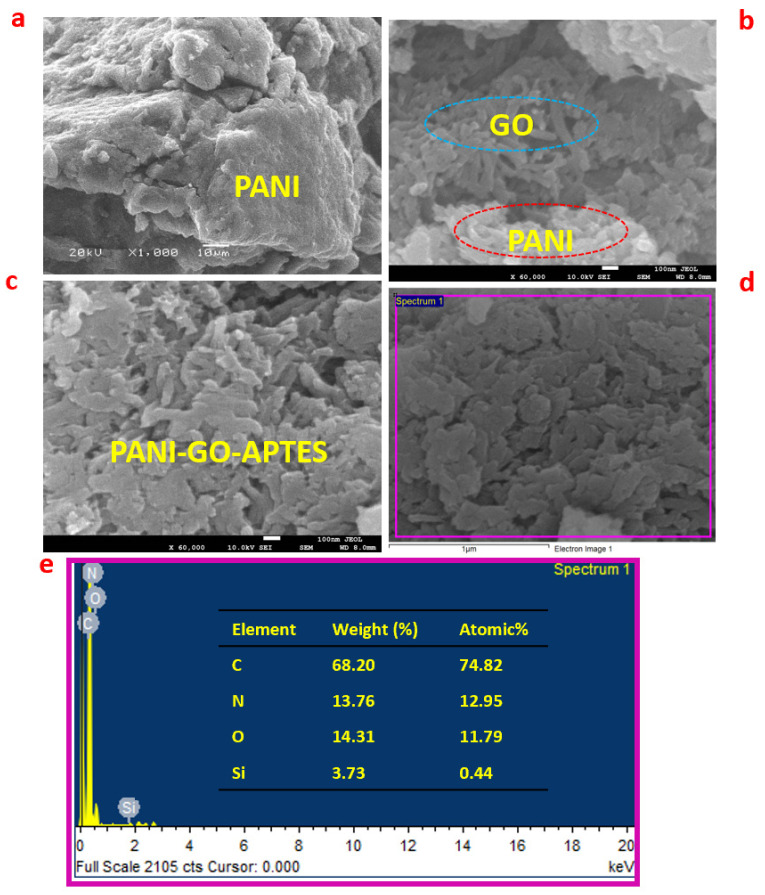
(**a**) SEM image of polyaniline. (**b**) SEM image showing grafting of GO on PANI substrate. (**c**) The magnified SEM image of PANI-GO. (**d**) SEM image of PANI-GO-APTES. (**e**) XEDS of PANI-GO-APTES.

The obtained SEM image results are presented in Figure 1. At first, PANI was synthesized, and the obtained image in Figure 1a reveals the formation of an amorphous structure typical for PANI [40]. Upon doping with GO, the crystalline nature of GO brought about improved structural image for PANI-GO, as displayed in Figure 1b. It can be observed that the PANI acts as a filler for the GO. However, upon doping with APTES, the crystallinity of the polymeric composite disappeared (Figure 1c). The obtained changes in the images in Figure 1 are an indication of the composition of different materials at each stage. 

#### 3.1.2. Elemental Composition Investigation with XEDS and XPS

The XEDS spectrum obtained for PANI-GO-APTES is shown in Figure 1e. The composite is composed of carbon, oxygen, nitrogen and Si. The listed elements belong to the expected elemental constituents of PANI-GO-APTES.

**Figure 2:**
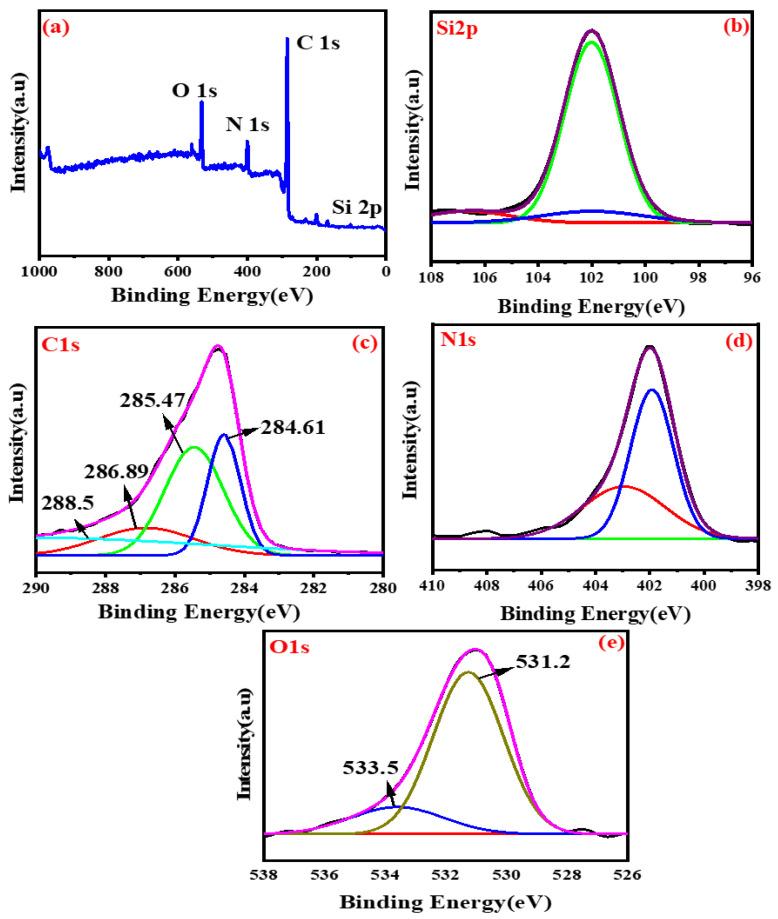
(**a**) Full XPS spectrum of PANI-GO-APTES. (**b**) Deconvoluted peak of Si. (**c**) Deconvoluted peak of carbon. (**d**) Deconvoluted peak of nitrogen. (**e**) Deconvoluted peak of oxygen.

In another effort, XPS was carried out to determine the binding energy of the material under study (PANI-GO-APTES). XPS is a powerful tool for surface chemical investigation. The full XPS spectrum recorded for PANI-GO-APTES (Figure 2a) indicates that four major elements are present in the as-prepared PANI-GO-APTES. These elements are silicon, carbon, nitrogen and oxygen. The constituent elements were deconvoluted to reveal all types of bonding interaction in the composite. The Si peak at 102 eV is associated with Si-O-C bond, as presented in Figure 2b [41].

Carbon is deconvoluted into four peaks, namely C-O-C/C-O-Si, C-C/C=C, C-N and C=O corresponding to 286.89, 284.6, 285.47 and 288.5 eV, respectively [41], as presented in Figure 2c.

The nitrogen spectra are deconvoluted into two peaks, namely C-N and H-N corresponding to the peaks at 401.9 and 403 eV, respectively (Figure 2d). Moreover, oxygen is deconvoluted into C=O and Si-O peaks corresponding to peaks at 531.2 and 533.5 eV, respectively (Figure 2e) [41]. The obtained XPS results are highly suggestive of successful synthesis of PANI-GO-APTES.

#### 3.1.3. Structural Investigation

**Figure 3:**
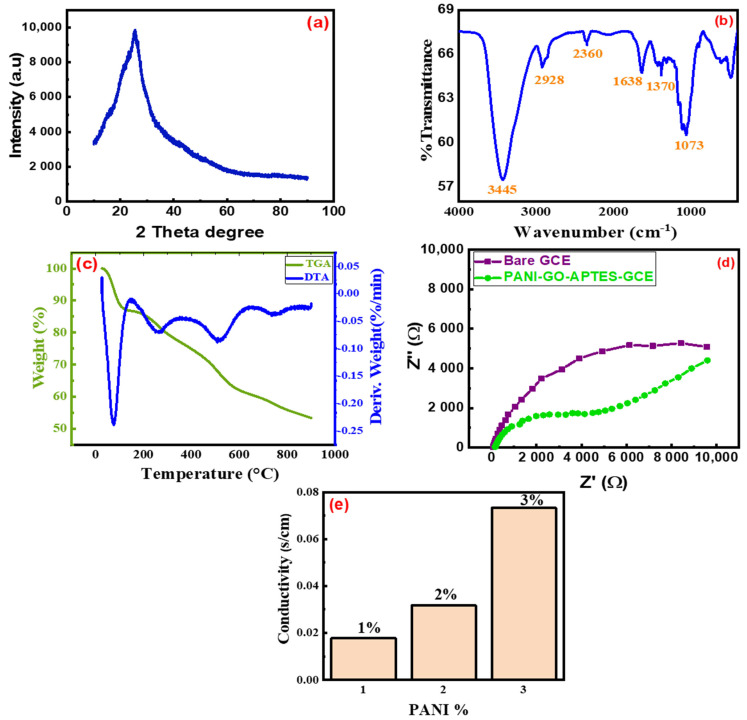
(**a**) XRD spectrum recorded for PANI-GO-APTES. (**b**) FTIR spectrum recorded for PANI-GO-APTES. (**c**) Recorded TGA and DTA spectra. (**d**) Recorded EIS spectrum in potassium ferricyanide. (**e**) Recorded electrical conductivity values for GO/APTES nanocomposite with PANI loading of 1%, 2% and 3% at room temperature.

The structural investigation of the synthesized PANI-GO-APTES was studied with XRD. The obtained XRD spectrum is presented in Figure 3a. The image reveals a noncrystalline material. The diffraction peak at 26.6 °C is typical for GO, while the small peak at 28° could be due to the incorporation of PANI into the composite [42,43]. The peaks have low diffraction because of the noncrystalline nature of the composite. 

#### 3.1.4. FTIR Analysis

Figure 3b presents the FTIR spectrum obtained for the PANI-GO-APTES composite. The intense absorption peak at 3445 cm^−1^ corresponds to N-H vibration of PANI and APTES. In addition, the peaks at 1638 and 1370 cm^−1^ could be attributed to quinoid and benzenoid vibrations, which implies effective interaction between GO-APTES and PANI. The observed peak at 1630 cm^−1^ may be due to N-H stretching vibration in PANI and APTES. Interaction between APTES and GO could bring about Si-O-Si stretching vibration, resulting in the peak at around 1097 cm^−1^. This peak could imply successful anchoring of the Si of APTES on the GO surface. The C-H stretching of APTES, PANI and GO could be associated with the peaks at 2928 and 2360 cm^−1^. The obtained results are consistent with the literature [19,44,45].

#### 3.1.5. Thermal Stability Investigation

The obtained TGA spectrum for the as-prepared PANI-GO-APTES is presented in Figure 3c. The spectrum shows that at 200 °C, the composite started to lose about 12% of its mass. This weight loss is associated with the adsorbed water molecules on the composite’s surface being expelled. From 200 to 850 °C, 75% of the mass was lost due to the decomposition of the organic component of the composite. The result showed that the prepared composite has good thermal stability up to the ashing temperature. Three major derivative peaks were found on the DTA spectrum, observed at 200, 400 and 800 °C. The obtained result suggests an exothermic reaction during the content phase.

#### 3.1.6. Electrochemical Impedance Spectroscopy (EIS)

The EIS spectrum recorded for PANI-GO-APTES in 1 mM ferricyanide is presented in Figure 3d. In EIS analysis, the charge transfer resistance, Rct, is denoted by the semicircle of the Nyquist plot. A smaller value of Rct implies low resistance and improved charge transfer on the electrode surface. The Rct for PANI-GO-APTES was found to be 2980 ohms, while that of unmodified GCE was 10,100 ohms. The obtained result indicates that coating PANI-GO-APTES on the GCE surface greatly improved the charge transfer resistance on the electrode surface, thereby reducing the current resistance. This property is also thought to contribute to the catalytic ability of PANI-GO-APTES in electroreduction reaction of Pb^2+^ to zerovalent Pb. The circuit diagram for the Nyquist plot and the values for the circuit parameters are presented in supporting information (Figure S1 and Figure S2).

#### 3.1.7. Electrical Conductivity Using Four-Probe Method

The electrical resistivity of the as-prepared sample was measured using the four-probe method. The obtained current–voltage data were used to measure electrical resistivity using the following equations (Equations (1)–(4)) [46]:

(1) $$\rho =\rho ˳∕G_{7}\;(W/S)$$

where ρ˳ is uncorrected resistivity (ohm cm); ρ is corrected resistivity (ohm cm); and G_7_ (W/S) is the correction factor used for the case of a nonconducting bottom surface, which is a function of W, S the probe spacing (cm) and the thickness of the sample under test (cm).

(2) $$G_{7}∕(W/S)=\left(2S∕W\right)\;ln2$$

(3) ρ˳= V/I×2πS

where I is the current (A), σ is DC electrical conductivity (S cm^−1^) and V is the voltage (V).

(4) $$\sigma =\frac{1}{\rho }$$

The obtained results are summarized in Figure 3e. The composite containing 3% PANI displayed the highest conductivity, followed by that containing 2%. The least conductive material was that containing 1% PANI, which shows that PANI greatly controlled the conductivity of the samples. The composites using higher PANI contents had lower electrical resistivity at 13.65 (Ω cm) and higher stability according to the formula described in Table S1.

### 3.2. Application of PANI-GO-APTES as Sensor for Pb^2+^ in Aqueous Solution

#### 3.2.1. Selectivity

**Figure 4:**
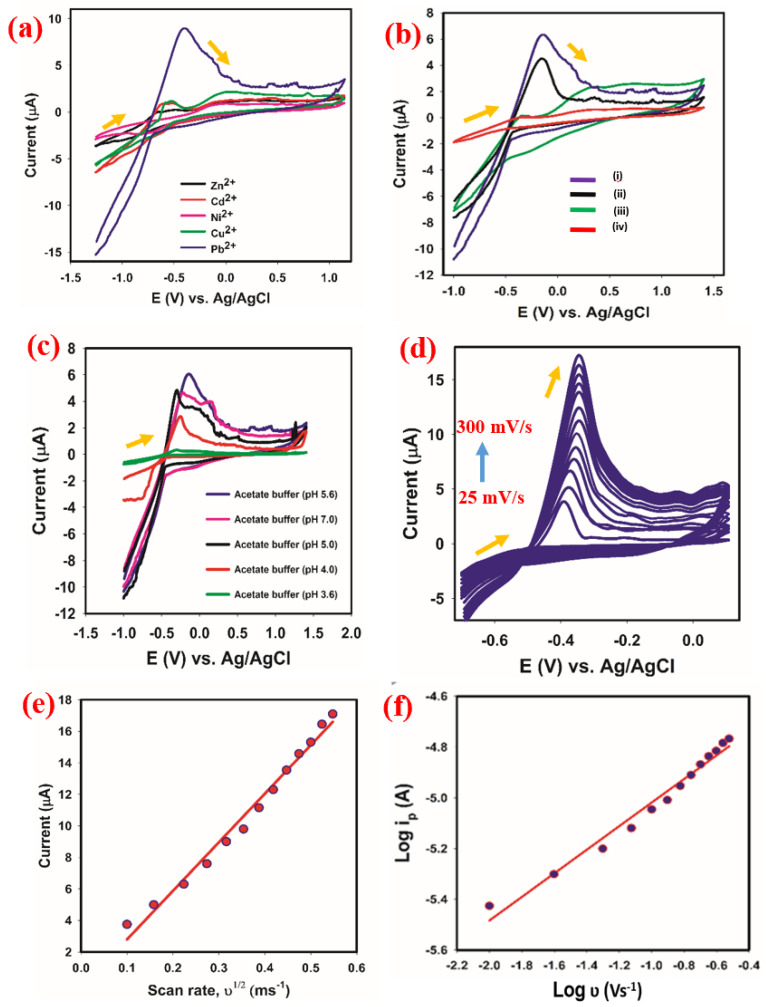
(**a**) The cyclic voltammogram recorded for selectivity study in 1 µM of the analytes. (**b**) The cyclic voltammogram obtained for control study: (i) PANI-GO-APTES_AcB + 0.5 µM Pb^2+^; (ii) GO-APTES_AcB + 0.5 µM Pb^2+^; (iii) PANI-GO-APTES_AcB only; (iv) bare GCE_AcB only. (**c**) The cyclic voltammogram obtained in acetate buffer containing 0.5 µM Pb^2+^ in different pH media. (**d**) The cyclic voltammogram obtained at different scan rates—25 to 300 mV/s. (**e**) Plot of reduction peak current versus square root of the scan rate. (**f**) Plot of log of peak current versus log of scan rate.

Figure 4a presents the current response of PANI-GO-APTES to different metal ions at the same concentration. PANI-GO-APTES responded selectively to 1 µM Pb^2+^ with a distinguished high reduction current at −0.4 V. Other metal ions such as Ni^2+^, Cd^2+^, Zn^2+^ and Cu^2+^ had very little or no reduction current at this potential. Therefore, it was concluded that PANI-GO-APTES is sensitive and selective towards Pb^2+^. Hence, further electrochemical detection studies were carried out for Pb^2+^.

#### 3.2.2. Control Experiment

Figure 4b presents the current response of PANI-GO-APTES composite, GO-APTES composite and bare glassy carbon electrode in 0.5 µM Pb^2+^. From the figure, it can be observed that PANI-GO-APTES displayed a distinguished high reduction current at −0.4 V. As compared to GO-APTES composite, the reduction current was higher, and this implies that incorporation of PANI into GO-APTES improved the electroreduction/catalytic property of GO-APTES. As reported elsewhere, PANI has excellent catalytic properties, which makes its use in electrochemical sensing very promising [47,48,49]. On the other hand, unmodified GCE did not present current response towards Pb^2+^. Equally, PANI-GO-APTES did not present reduction current at −0.4 V in the absence of Pb^2+^ (acetate buffer only). From this control study, it is established that the observed reduction current at −0.4 V was due to PANI-GO-APTES.

#### 3.2.3. Influence of pH of Supporting Electrolyte

Figure 4c presents the recorded current response of PANI-GO-APTES in the solution containing acetate buffer (of different pH media) and Pb^2+^. The optimum reduction current was recorded with acetate buffer of pH 5.6. This suggests that electroreduction of Pb^2+^ was more favorable in an acidic buffer medium. On the other hand, in the alkaline medium (result not included), a viscous white precipitate was immediately formed in the electrochemical cell upon spiking with Pb^2+^. This resulted in no or very low current response. Therefore, the pH of the supporting electrolyte was maintained in the range of 7.0 to 3.6. From the result, the lowest reduction current was observed with pH 5.6. Therefore, the electrochemical studies for Pb^2+^ detection were conducted using the optimum pH medium (pH 5.6).

#### 3.2.4. Influence of Scan Rate

Scan rate gives vital information on the electrochemical behavior of an analyte on the electrode surface [50]. For instance, the scan rate gives information about the diffusivity or absorptivity of the reaction. The cyclic voltammogram obtained at varying scan rates (from 25 to 300 mV/s) is given in Figure 4d. In Figure 4e, the plot of the reduction current peaks against the square root of scan rate is displayed. A linear response was observed between the two parameters, suggesting a diffusion-controlled reaction. The equation of the plot is given in Equation (5) with a correlation of 0.99.

i_p_ = 30.82 × V^1/2^ − 0.29       (R^2^ = 0.99) (5)

For confirmation of the nature of the electrochemical reaction occurring on the electrode surface, the plot of the logarithm of reduction current response against the logarithm of scan rate was plotted (Figure 4f). The equation of the plot is presented in Equation (6).

Log i_p_ = 0.46 × Log V − 4.55       (R^2^ = 0.98) (6)

As given in Equation (1), the slope of the plot is 0.46; slopes of <0.5 indicate a fully diffusion-controlled reaction, while those >0.5 indicate adsorption-controlled reactions [51]. For the current study, the observed slope was 0.46, which confirms the reaction is diffusion-controlled.

### 3.3. Influence of Varying Pb^2+^ Concentration

**Figure 5:**
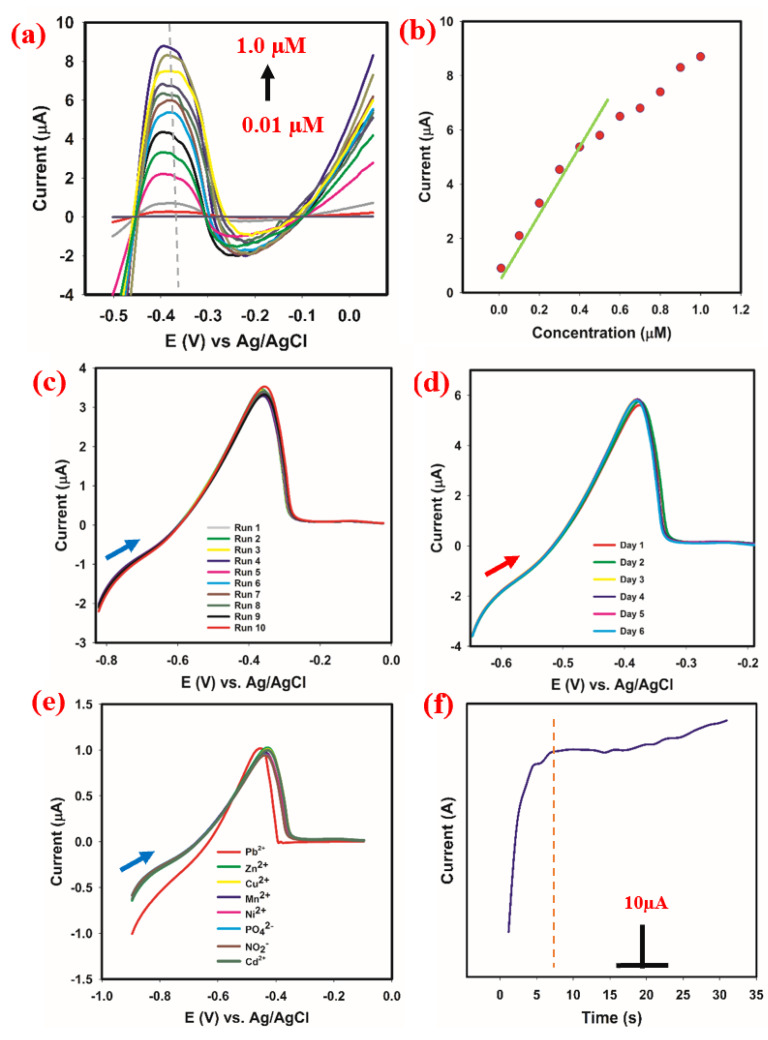
(**a**) Calibration plot using linear sweep voltammetry with PANI-GO-APTES-modified GCE as working electrode. (**b**) Plot of peak current versus concentration of Pb^2+^. (**c**) The recorded linear sweep voltammogram in 10 successive runs in 0.2 µM. (**d**) The recorded linear sweep voltammogram using different GCEs modified with PANI-GO-APTES in 0.2 µM. (**e**) The obtained linear sweep voltammogram for Pb^2+^ in the presence of added metals (interferents) in 0.01 µM. (**f**) Response time for PANI-GO-APTES in 100 nM Pb^2+^ in 0.2 µM.

The recorded current response of PANI-GO-APTES towards varying concentrations of Pb^2+^ is presented in Figure 5a. Before the addition of Pb^2+^, no reduction peak was observed at −0.4 V. However, upon addition of 0.01 µM Pb^2+^, a reduction peak was observed. The peak increased linearly up to 0.4 µM. The plot of varying Pb^2+^ concentration against the reduction current is presented in Figure 5b, and the equation of the plot is given in Equation (7).

i_p_ = 11.60 × C (µM) + 0.89 (R^2^ = 0.99) (7)

The obtained correlation (0.99) suggests a good linear response in the linear range of 0.01 to 0.4 µM. The mechanism of detection of Pb^2+^ on the surface of PANI-GO-APTES might be due to electroreduction of Pb^2+^ to zerovalent Pb catalyzed by PANI-GO-APTES substrate. The reaction is illustrated in Equation (8):

(8) $${\mathrm{Pb}}^{2+}+2e^{-}\to \mathrm{Pb}$$

### 3.4. Analytical Performance of PANI-GO-APTES in the Detection of Pb^2+^

#### 3.4.1. Parameters of Merit

The merit parameters of interest for this study are limit of detection (LOD), limit of quantification (LOQ), sensitivity, linear dynamic range (LDR) and full dynamic range (FDR). The LOD, LOQ and sensitivity were calculated using the formula given in Parameter S1. These parameters were calculated based on the linear current response range of the calibration. The obtained values for LOD, LOQ and sensitivity were 0.0053 µM, 0.017 µM and 165.71 µA µM cm^−1^ respectively. Moreover, the linear dynamic range and full dynamic range for the calibration plot were 0.01 to 0.4 µM and 0.01 to 1.0 µM, respectively.

#### 3.4.2. Sensor’s Stability

The stability of PANI-GO-APTES-modified GCE for Pb^2+^ detection was examined by repeatability, reproducibility, interference studies and response time. Figure 5c presents the reduction current obtained in 10 consecutive runs maintaining the same experimental conditions. The relative standard deviation (RSD) of the recorded peak reduction current was 2.1%. In addition, Figure 5d presents the obtained results with differently prepared PANI-GO-APTES-modified GCEs in the same Pb^2+^ concentration. The RSD of the recorded reduction peak current was 1.5%. 

The probable effect of likely interferents such as Ni^2+^, Cu^2+^, Mn^2+^, Zn^2+^, Cd^2+^ and other electroactive species such as nitrite and phosphate ions was investigated, and the obtained result is given in Figure 5e. The reduction peak current displayed by PANI-GO-APTES was stable and consistent even with the addition of the mentioned interferents. The RSD of the reduction current response was 1.2%, and the peak current ranged from 0.89 to 1.01 µA (Figure 5e). The results obtained in the stability study indicate that the current response of PANI-GO-APTES towards Pb^2+^ is very repeatable, reproducible and interference-buffered.

In addition, the response time of PANI-GO-APTES in Pb^2+^ detection is presented in Figure 5f. The current response attains some stability at about 7 s. This shows that PANI-GO-APTES could be suitable for in situ detection of Pb^2+^ as it has a short response time desirable for in situ detector/sensors.

### 3.5. Application of the Proposed Sensor Based on PANI-GO-APTES for Real Sample Analysis

The real samples used for this study were tap water, well water and bottled water. A standard addition method of spiking the electrochemical cell with a known amount of Pb^2+^ was employed. The percentage recovery of the spiked Pb^2+^ was then estimated using the formula stated in Parameter S2. The obtained result is presented in Table 1. From the obtained results, the percentage recovery of spiked Pb^2+^ ranged from 86.7 to 106.7%. The obtained result suggests that PANI-GO-APTES can be used for the real detection of Pb^2+^.

### 3.6. Performance Comparison with Previous Work

As indicated in Section 1, several attempts have been made to develop cheap and efficient sensors for detecting Pb^2+^ in the environment due to its medical importance. Some of the pertinent work related to Pb^2+^ detection is displayed in Table 2. PANI-GO-APTES displayed superior performance when compared to most of the reported results.

## 4. Conclusions

In this study, novel PANI-GO-APTES composite was synthesized by wet chemical method, and its formation was confirmed using characterization techniques such as X-ray diffraction spectroscopy, scanning electron microscopy, X-ray photoelectron spectroscopy, thermal gravimetry analysis, electrochemical impedance spectroscopy and cyclic voltammetry. The characterization studies revealed that the synthesized PANI-GO-APTES had an amorphous structure, good electrical conductivity and good thermal stability. Due to its excellent electrochemical property, PANI-GO-APTES was investigated for electrochemical sensing application. PANI-GO-APTES cast on the glassy carbon electrode responded to Pb^2+^ selectively using cyclic and linear sweep voltammetry methods. The minimum detectable concentration of lead was 0.0053 µM in the linear range of 0.01 to 0.4 µM. The sensitivity recorded was 165.71 µA µM^−1^cm^−1^, and the response time was less than 20 s. In the presence of likely interferents such as metal ions and electroactive species, PANI-GO-APTES current response towards Pb^2+^ was stable. The proposed method was also successfully applied for Pb^2+^ detection in real environmental water samples (tap, well and bottled water) with good percentage recovery. Therefore, this study established the application of PANI-GO-APTES-modified GCE for electrochemical detection of lead with good performance. The outcome of this research is a step in achieving a cheap, sensitive and accurate method for detection of lead in the environment to improve human health and protect the environment.

## Figures and Tables

**Table 1:** The result of real sample analysis.

Sample	NS *	Spiked Concentration (µM)	Found Concentration (µM)	Bias	Recovery (%)	RSD (%)
Tap water	3	0	0	-	-	-
	3	0.03	0.032 + 0.0011	+0.002	106.67%	3.4
	3	0.2	0.19 + 0.017	−0.01	95%	8.9
Well water	3	0	0	-	-	-
	3	0.03	0.026 + 0.002	+0.004	86.67%	7.69
	3	0.2	0.206 + 0.003	+0.006	103%	1.46
Bottled water	3	0	0	-	-	-
	3	0.2	0.19 + 0.012	−0.01	95%	6.32

* NS—number of samples analyzed.

**Table 2:** Comparison of current research work with some of the previous work.

Electrode/Substrate	LDR µM	Method	LOD µM	Ref.
GCE/CoTBrImPPc	0.1–1	CV	0.03	[52]
TiO_2_/rGO/GCE	1 × 10^−5^–1 × 10^−4^	DPV	0.03	[53]
MnFe_2_O_4_/GO GCE	0.2–1.1	SWASV	0.0883	[54]
AuNS	0.188–0.65	SWASV	0.062	[55]
AgNPs/RGO/GCE	0.05–2.5	SWASV	0.155	[56]
GC/ErGO	0.045–0.317	SWASV	0.045	[57]
Cu based MOFs/GCE	0.01–1000	DPV	0.05	[58]
Alk-Ti3C_2_/GCE	0.1–1.5	SWASV	0.041	[59]
BTC-GCE	7 × 10^−3^–9 × 10^2^	SWASV	44.08	[60]
Functionalized Au-NPs	100–1000	Colorimetry	1.8	[61]
Amino-carbon dots/Au-NPs	2–60	Fluorescence	0.5	[62]
PANI@APTES-GO/Nafion/GCE	0.01–0.4 µM	LSV	0.0053	Current study

## Supplementary Materials

The following are available online at https://www.mdpi.com/article/10.3390/polym13152562/s1, Figure S1: EIS spectrum for PANI-MWCNT-APTES modified GCE. Where Rct denotes charge transfer resistance; Rs denotes solution resistance; CPE denotes constant phase element, W denotes Warbug impedance and C denotes capacitance, Figure S2: EIS spectrum for bare GCE, Table S1: Electrical resistivity, Parameter S1: Analytical performance parameters, Parameter S2: Real sample analysis.

## References

[1] Khan A., Khan A.A.P., Asiri A.M., Gupta V., Rathore M. (2016). Preparation, properties and applications of organic–inorganic hybrid nanocomposite poly(aniline-co-o-toluidine) tungstomolybdate. J. Mol. Liq..

[2] Usuki A., Kojima Y., Kawasumi M., Okada A., Fukushima Y., Kurauchi T., Kamigaito O. (1993). Synthesis of nylon 6-clay hybrid. J. Mater. Res..

[3] Al Zoubi W., Kamil M.P., Fatimah S., Nashrah N., Ko Y.G. (2020). Recent advances in hybrid organic-inorganic materials with spatial architecture for state-of-the-art applications. Prog. Mater. Sci..

[4] Lee B.H., Yoon B., Abdulagatov A.I., Hall R.A., George S.M. (2013). Growth and Properties of Hybrid Organic-Inorganic Metalcone Films Using Molecular Layer Deposition Techniques. Adv. Funct. Mater..

[5] Meng X. (2017). An overview of molecular layer deposition for organic and organic–inorganic hybrid materials: Mechanisms growth characteristics and promising applications. J. Mater. Chem. A.

[6] Gogoi K.K., Chowdhury A. (2020). Performance Enhancement of Solution-Processed Organic Memories by Exploiting Synergistic Organic–Inorganic Hybrid Composites. J. Phys. Chem. C.

[7] Ul Haque S., Nasar A., Rahman M.M. (2020). Applications of chitosan (CHI)-reduced graphene oxide (rGO)-polyaniline (PAni) conducting composite electrode for energy generation in glucose biofuel cell. Sci. Rep..

[8] Ke F., Liu Y., Xu H., Ma Y., Guang S., Zhang F., Lin N., Ye M., Lin Y., Liu X. (2017). Flower-like polyaniline/graphene hybrids for high-performance supercapacitor. Compos. Sci. Technol..

[9] Gahlot S., Kulshrestha V. (2020). Graphene based polymer electrolyte membranes for electro-chemical energy applications. Int. J. Hydrogen Energy.

[10] Tiwari S., Purabgola A., Kandasubramanian B. (2020). Functionalised graphene as flexible electrodes for polymer photovoltaics. J. Alloys Compd..

[11] Bonaccorso F., Sun Z., Hasan T., Ferrari A.C. (2010). Graphene photonics and optoelectronics. Nat. Photonics.

[12] Bouville F., Maire E., Meille S., Van de Moortèle B., Stevenson A.J., Deville S. (2014). Strong, tough and stiff bioinspired ceramics from brittle constituents. Nat. Mater..

[13] Khan A., Parwaz Khan A.A., Khan I., Oves M., Khan S., Asiri A.M., Azum N., Taib L.A., Al Angari Y.M., Facchetti A. (2019). Facial synthesis of highly active polymer vanadium molybdate nanocomposite: Improved thermoelectric and antimicrobial studies. J. Phys. Chem. Solids.

[14] Lingamdinne L.P., Koduru J.R., Karri R.R. (2019). A comprehensive review of applications of magnetic graphene oxide based nanocomposites for sustainable water purification. J. Environ. Manag..

[15] Haque S.U., Nasar A., Asiri A.M. (2019). Preparation and characterization of a bioanode (GC/MnO 2 /PSS/Gph/Frt/GOx) for biofuel cell application. Int. J. Hydrogen Energy.

[16] Han Y., Qian C., Wu H., Chen X., Wu X., He W., Yan H., Li G., Diao G., Chen M. (2021). Two flowers per seed: Derivatives of CoG@F127/GO with enhanced catalytic performance of overall water splitting. J. Energy Chem..

[17] Sonawane S., Thakur P., Paul R. (2020). Study on thermal property enhancement of MWCNT based polypropylene (PP) nanocomposites. Mater. Today Proc..

[18] Peng B., Takai C., Razavi-khosroshahi H., Fuji M. (2018). Effect of silane modification on CNTs/silica composites fabricated by a non-firing process to enhance interfacial property and dispersibility. Adv. Powder Technol..

[19] Zhi X., Mao Y., Yu Z., Wen S., Li Y., Zhang L., Chan T.W., Liu L. (2015). γ-Aminopropyl triethoxysilane functionalized graphene oxide for composites with high dielectric constant and low dielectric loss. Compos. Part A Appl. Sci. Manuf..

[20] Frank J.J., Poulakos A.G., Tornero-Velez R., Xue J. (2019). Systematic review and meta-analyses of lead (Pb) concentrations in environmental media (soil, dust, water, food, and air) reported in the United States from 1996 to 2016. Sci. Total Environ..

[21] Li C.-L., Liu K.-T., Lin Y.-W., Chang H.-T. (2011). Fluorescence Detection of Lead(II) Ions Through Their Induced Catalytic Activity of DNAzymes. Anal. Chem..

[22] Yan M., Zhu C., Huang Y., Yan J., Chen A. (2017). Ultrasensitive detection of lead(II) using a turn-on probe based on the use of an aptamer and a water-soluble fluorescent perylene probe. Microchim. Acta.

[23] Sivasubramanian R., Sangaranarayanan M. (2011). V Detection of lead ions in picomolar concentration range using underpotential deposition on silver nanoparticles-deposited glassy carbon electrodes. Talanta.

[24] Liu W.-Y., Ju X.-J., Faraj Y., He F., Peng H.-Y., Liu Y.-Q., Liu Z., Wang W., Xie R., Chu L.-Y. (2020). Capsule membranes encapsulated with smart nanogels for facile detection of trace lead(II) ions in water. J. Membr. Sci..

[25] Duong T.D.S., Jang C.-H. (2020). A label-free liquid crystal droplet-based sensor used to detect lead ions using single-stranded DNAzyme. Colloids Surf. A Physicochem. Eng. Asp..

[26] Chu J., Chen C., Li X., Yu L., Li W., Cheng M., Tang W., Xiong Z. (2021). A responsive pure DNA hydrogel for label-free detection of lead ion. Anal. Chim. Acta.

[27] Chabbah T., Abderrazak H., Souissi R., Saint-Martin P., Casabianca H., Chatti S., Mercier R., Rassas I., Errachid A., Hammami M. (2020). A Sensitive Impedimetric Sensor Based on Biosourced Polyphosphine Films for the Detection of Lead Ions. Chemosensors.

[28] Kim R., Youn Y.-S., Kang M., Kim E. (2020). Platform- and label-free detection of lead ions in environmental and laboratory samples using G-quadraplex probes by circular dichroism spectroscopy. Sci. Rep..

[29] Chen G., Bai W., Jin Y., Zheng J. (2021). Fluorescence and electrochemical assay for bimodal detection of lead ions based on Metal–Organic framework nanosheets. Talanta.

[30] Martín-Yerga D., Álvarez-Martos I., Blanco-López M.C., Henry C.S., Fernández-Abedul M.T. (2017). Point-of-need simultaneous electrochemical detection of lead and cadmium using low-cost stencil-printed transparency electrodes. Anal. Chim. Acta.

[31] Baghayeri M., Ghanei-Motlagh M., Tayebee R., Fayazi M., Narenji F. (2020). Application of graphene/zinc-based metal-organic framework nanocomposite for electrochemical sensing of As(III) in water resources. Anal. Chim. Acta.

[32] Wei P., Zhu Z., Song R., Li Z., Chen C. (2019). An ion-imprinted sensor based on chitosan-graphene oxide composite polymer modified glassy carbon electrode for environmental sensing application. Electrochim. Acta.

[33] Sreenivasa Rao K., Balaji T., Prasada Rao T., Babu Y., Naidu G.R.K. (2002). Determination of iron, cobalt, nickel, manganese, zinc, copper, cadmium and lead in human hair by inductively coupled plasma-atomic emission spectrometry. Spectrochim. Acta Part B At. Spectrosc..

[34] Daşbaşı T., Saçmacı Ş., Çankaya N., Soykan C. (2016). A new synthesis, characterization and application chelating resin for determination of some trace metals in honey samples by FAAS. Food Chem..

[35] Munir A., Shah A., Nisar J., Ashiq M.N., Akhter M.S., Shah A.H. (2019). Selective and simultaneous detection of Zn^2+^, Cd^2+^, Pb^2+^, Cu^2+^, Hg^2+^ and Sr^2+^ using surfactant modified electrochemical sensors. Electrochim. Acta.

[36] Rahman N.H.B.T., Tee T.W., Sirat K. (2011). Adsorption enhancement of Pb(II) ion in the presence of Nicotinic acid during cyclic Voltammetry. Int. J. Electrochem. Sci..

[37] Riyanto D. (2015). Determination of lead in waste water using cyclic voltammetry by platinum wire electrode. Eksakta J. Ilmu-Ilmu MIPA.

[38] Magerusan L., Socaci C., Coros M., Pogacean F., Rosu M.C., Gergely S., Pruneanu S., Leostean C., Pana I.O. (2017). Electrochemical platform based on nitrogen-doped graphene/chitosan nanocomposite for selective Pb^2+^ detection. Nanotechnology.

[39] Anambiga I.V., Suganthan V., Raj N.A.N., Kumar A.S. Electrochemical sensor for the detection of lead ions. Proceedings of the International Conference on Advanced Nanomaterials and Emerging Engineering Technologies.

[40] Ibrahim N.I., Wasfi A.S. (2019). A comparative study of polyaniline/MWCNT with polyaniline/SWCNT nanocomposite films synthesized by microwave plasma polymerization. Synth. Met..

[41] Huang J., Ding S., Xiao W., Peng Y., Deng S., Zhang N. (2015). 3-Aminopropyl-triethoxysilane functionalized graphene oxide: A highly efficient and recyclable catalyst for knoevenagel condensation. Catal. Lett..

[42] Yan S., Yang Y., Song L., Qi X., Xue Y., Fan B. (2017). Influence of 3-aminopropyltriethoxysilane- graphite oxide composite on thermal stability and mechanical property of polyethersulfone. High Perform. Polym..

[43] Ma W., Wu L., Zhang D., Wang S. (2013). Preparation and properties of 3-aminopropyltriethoxysilane functionalized graphene/polyurethane nanocomposite coatings. Colloid Polym. Sci..

[44] Etemadi M., Samadi S., Yazd S.S., Jafari P., Yousefi N., Aliabadi M. (2017). Selective adsorption of Cr(VI) ions from aqueous solutions using Cr6+-imprinted Pebax/chitosan/GO/APTES nanofibrous adsorbent. Int. J. Biol. Macromol..

[45] Guler M., Dilmac Y. (2019). Palladium nanoparticles decorated (3-aminopropyl)triethoxysilane functionalized reduced graphene oxide for electrochemical determination of glucose and hydrogen peroxide. J. Electroanal. Chem..

[46] Abu-Zied B.M., Hussein M.A., Khan A., Asiri A.M. (2018). Cu-Cu2O@graphene nanoplatelets nanocomposites: Facile synthesis, characterization, and electrical conductivity properties. Mater. Chem. Phys..

[47] Ourari A., Zerdoumi R., Ruiz-Rosas R., Morallon E. (2019). Synthesis and catalytic properties of modified electrodes by pulsed electrodeposition of Pt/PANI nanocomposite. Materials.

[48] Mozafari V., Parsa J.B. (2020). Electrochemical synthesis of Pd supported on PANI-MWCNTs-SnO2 nanocomposite as a novel catalyst towards ethanol oxidation in alkaline media. Synth. Met..

[49] Soleimani-Lashkenari M., Rezaei S., Fallah J., Rostami H. (2018). Electrocatalytic performance of Pd/PANI/TiO2 nanocomposites for methanol electrooxidation in alkaline media. Synth. Met..

[50] Adeosun W.A., Asiri A.M., Marwani H.M., Rahman M.M. (2020). Enzymeless Electrocatalytic Detection of Uric Acid Using Polydopamine/Polypyrrole Copolymeric film. Chem. Sel..

[51] Adeosun W.A., Asiri A.M., Marwani H.M. (2020). Sensitive determination of 2-nitrophenol using electrochemically deposited polymethyl red film for healthcare and environmental safety. Synth. Met..

[52] Sajjan V.A., Aralekallu S., Nemakal M., Palanna M., Prabhu C.P.K., Sannegowda L.K. (2020). Nanomolar detection of lead using electrochemical methods based on a novel phthalocyanine. Inorg. Chim. Acta.

[53] Veera Manohara Reddy Y., Sravani B., Łuczak T., Mallikarjuna K., Madhavi G. (2021). An ultra-sensitive rifampicin electrochemical sensor based on titanium nanoparticles (TiO_2_) anchored reduced graphene oxide modified glassy carbon electrode. Colloids Surf. A Physicochem. Eng. Asp..

[54] Zhou S.-F., Han X.-J., Fan H.-L., Huang J., Liu Y.-Q. (2018). Enhanced electrochemical performance for sensing Pb(II) based on graphene oxide incorporated mesoporous MnFe2O4 nanocomposites. J. Alloys Compd..

[55] Dutta S., Strack G., Kurup P. (2019). Gold nanostar electrodes for heavy metal detection. Sens. Actuators B Chem..

[56] Sang S., Li D., Zhang H., Sun Y., Jian A., Zhang Q., Zhang W. (2017). Facile synthesis of AgNPs on reduced graphene oxide for highly sensitive simultaneous detection of heavy metal ions. RSC Adv..

[57] Pizarro J., Segura R., Tapia D., Bollo S., Sierra-Rosales P. (2019). Electroanalytical Determination of Cd(II) and Pb(II) in Bivalve Mollusks using Electrochemically Reduced Graphene Oxide-based Electrode. Electroanalysis.

[58] Hu R., Zhang X., Chi K.-N., Yang T., Yang Y.-H. (2020). Bifunctional MOFs-Based Ratiometric Electrochemical Sensor for Multiplex Heavy Metal Ions. ACS Appl. Mater. Interfaces.

[59] Zhu X., Liu B., Hou H., Huang Z., Zeinu K.M., Huang L., Yuan X., Guo D., Hu J., Yang J. (2017). Alkaline intercalation of Ti3C2 MXene for simultaneous electrochemical detection of Cd(II), Pb(II), Cu(II) and Hg(II). Electrochim. Acta.

[60] Sultan S., Shah A., Khan B., Nisar J., Shah M.R., Ashiq M.N., Akhter M.S., Shah A.H. (2019). Calix[4]arene Derivative-Modified Glassy Carbon Electrode: A New Sensing Platform for Rapid, Simultaneous, and Picomolar Detection of Zn(II), Pb(II), As(III), and Hg(II). ACS Omega.

[61] Yu Y., Naik S.S., Oh Y., Theerthagiri J., Lee S.J., Choi M.Y. (2021). Lignin-mediated green synthesis of functionalized gold nanoparticles via pulsed laser technique for selective colorimetric detection of lead ions in aqueous media. J. Hazard. Mater..

[62] Wang L., Cao H.-X., He Y.-S., Pan C.-G., Sun T.-K., Zhang X.-Y., Wang C.-Y., Liang G.-X. (2019). Facile preparation of amino-carbon dots/gold nanoclusters FRET ratiometric fluorescent probe for sensing of Pb^2+^/Cu^2+^. Sens. Actuators B Chem..

