# Supplementary material for: Novel Aminosilane (APTES)-Grafted Polyaniline@Graphene Oxide (PANI-GO) Nanocomposite for Electrochemical Sensor
Source: Polymers (Basel). 2021 Jul 31;13(15):2562. doi: 10.3390/polym13152562 (PMC8347065; doi:10.3390/polym13152562)
Supplement: Supplementary file 1 [file polymers-13-02562-s001.zip › polymers-1315956-supplementary.pdf]

SUPPORTING INFORMATION

# Novel Aminosilane (APTES)-Grafted Polyaniline@Graphene Oxide (PANI-GO) Nanocomposite for Electrochemical Sensor

**Raja Saad Alruwais [1,2], Waheed A. Adeosun [1,3], Hadi M. Marwani [1,3], Mohammad Jawaid [4,*], Abdullah M. Asiri [1,3] and Anish Khan [1,3,*]**

[1] Chemistry Department, Faculty of Science, King Abdulaziz University, Jeddah 21589, Saudi Arabia;
rajaotb@gmail.com (R.S.A.); dsnwaheed1@gmail.com (W.A.A.);
hmarwani@kau.edu.sa (H.M.M.); aasiri2@kau.edu.sa (A.M.A.)
[2] Chemistry Department, Faculty of Science and Humanities, Shaqra University,
Dawadmi 11911, Saudi Arabia
[3] Center of Excellence for Advanced Materials Research, King Abdulaziz University, Jeddah 21589,
Saudi Arabia
[4] Department of Biocomposite Laboratory, Institute of Tropical Forestry and Forest Products (INTROP),
University Putra Malaysia, UPM Serdang 43400, Selangor, Malaysia
* Correspondence: jawaid@upm.edu.my (M.J.); akrkhan@kau.edu.sa (A.K.)

## Table S1: Electrical resistivity

| PANI% | Electrical resistivity (Ω Cm) | Electrical conductivity (σ) |
|---|---|---|
| **1** | 56.008 | 0.0178 S/cm |
| **2** | 31.54 | 0.0317 S/cm |
| **3** | 13.65 | 0.0732 S/cm |

## S2 Electrochemical Impedance Spectroscopy

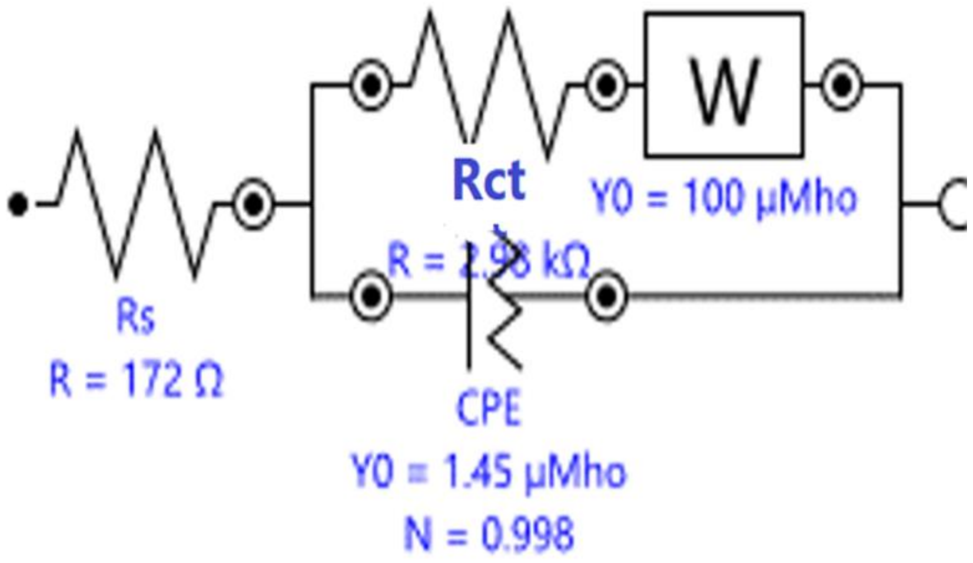


Figure S1. EIS spectrum for PANI-MWCNT-APTES modified GCE. Where Rct denotes charge transfer resistance; Rs denotes solution resistance; CPE denotes constant phase element, W denotes Warbug impedance and C denotes capacitance.

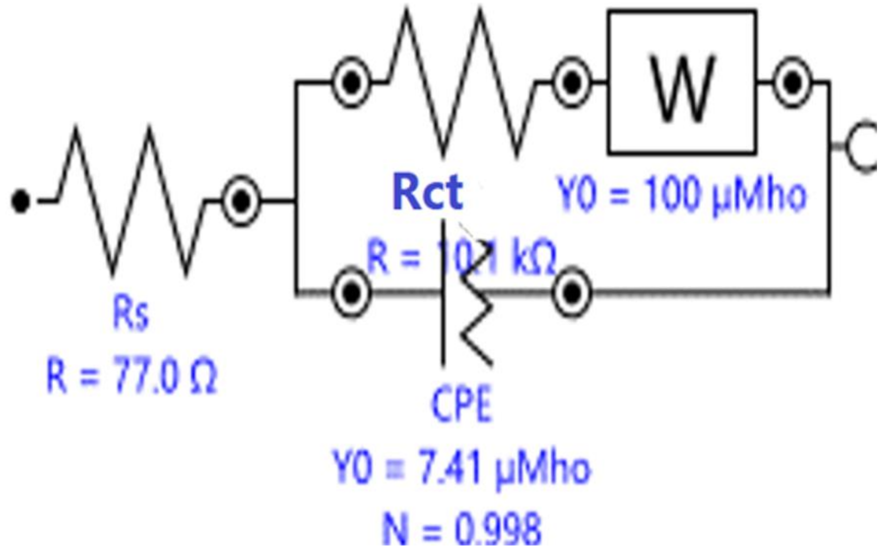


Figure S2. EIS spectrum for bare GCE.

# Parameter S1. Analytical performance parameters

***Limit of Detection (LOD)***

This was calculated using the equation;

LOD= 3 $\times$ Sd/b

where Sd is the standard deviation of the blank response and b is the calibration plot's slope.

***Limit of Quantification (LOQ)***

This was determined using the equation;

LOQ= 10 $\times$ Sd/b

## *Sensitivity*

This was calculated using the formula:

Sensitivity = slope of the calibration/ GCE surface area

# Parameter S2. Real sample analysis

Method used: Standard addition

Recovery (%) = $C_f/C_s \times 100$

Where $C_f$ is the found concentration of analyte, Cs is the concentration of the spiked Pb (II).
